# Aquaporin-4 autoantibodies in neuromyelitis optica spectrum disorders: comparison between tissue-based and cell-based indirect immunofluorescence assays

**DOI:** 10.1186/1742-2094-7-50

**Published:** 2010-09-07

**Authors:** Koon H Chan, Jason SC Kwan, Philip WL Ho, Jessica WM Ho, Andrew CY Chu, David B Ramsden

**Affiliations:** 1 University Department of Medicine, Queen Mary Hospital, LKS Faculty of Medicine, The University of Hong Kong, Hong Kong; 2 Neuroimmunology and Neuroinflammation Research Laboratory, LKS Faculty of Medicine, The University of Hong Kong, Hong Kong; 3 Research Center of Heart, Brain, Hormone and Healthy Aging, LKS Faculty of Medicine, The University of Hong Kong, Hong Kong

## Abstract

**Background:**

Neuromyelitis optica spectrum disorders (NMOSD) are severe central nervous system inflammatory demyelinating disorders (CNS IDD) characterized by monophasic or relapsing, longitudinally extensive transverse myelitis (LETM) and/or optic neuritis (ON). A significant proportion of NMOSD patients are seropositive for aquaporin-4 (AQP4) autoantibodies. We compared the AQP4 autoantibody detection rates of tissue-based indirect immunofluorescence assay (IIFA) and cell-based IIFA.

**Methods:**

Serum of Chinese CNS IDD patients were assayed for AQP4 autoantibodies by tissue-based IIFA using monkey cerebellum and cell-based IIFA using transfected HEK293 cells which express human AQP4 on their cell membranes.

**Results:**

In total, 128 CNS IDD patients were studied. We found that 78% of NMO patients were seropositive for AQP4 autoantibodies by cell-based IIFA versus 61% by tissue-based IFA (p = 0.250), 75% of patients having relapsing myelitis (RM) with LETM were seropositive by cell-based IIFA versus 50% by tissue-based IIFA (p = 0.250), and 33% of relapsing ON patients were seropositive by cell-based IIFA versus 22% by tissue-based IIFA (p = 1.000); however the differences were not statistically significant. All patients seropositive by tissue-based IIFA were also seropositive for AQP4 autoantibodies by cell-based IIFA. Among 29 NMOSD patients seropositive for AQP4 autoantibodies by cell-based IIFA, 20 (69%) were seropositive by tissue-based IIFA. The 9 patients seropositive by cell-based IIFA while seronegative by tissue-based IIFA had NMO (3), RM with LETM (3), a single attack of LETM (1), relapsing ON (1) and a single ON attack (1). Among 23 NMO or RM patients seropositive for AQP4 autoantibodies by cell-based IIFA, comparison between those seropositive (n = 17) and seronegative (n = 6) by tissue-based IIFA revealed no differences in clinical and neuroradiological characteristics between the two groups.

**Conclusion:**

Cell-based IIFA is slightly more sensitive than tissue-based IIFA in detection of AQP4 autoantibodies, which are highly specific for NMOSD.

## Background

Neuromyelitis optica (NMO) is a severe central nervous system inflammatory demyelinating disorder (CNS IDD) characterized by monophasic or relapsing optic neuritis (ON) and acute transverse myelitis (ATM) [1-6]. Relapsing NMO is the predominant type, which can mimic relapsing remitting multiple sclerosis (RRMS), the predominant form of classical multiple sclerosis (CMS), on initial presentation [2-8]. Typical relapsing NMO patients develop recurrent attacks of longitudinally extensive transverse myelitis (LETM) with MRI signal abnormalities extending over 3 or more spinal cord segments and severe ON resulting in frequent and early attack-related permanent disabilities and even mortality [2-6,8,9]. About 50% of patients require assistance with walking and 62% become functionally blind (visual acuity of 20/200 or worse) within 5 years of onset [2]; and ~15-30% die, predominantly from respiratory failure complicating high cervical myelitis, brainstem involvement and fatal dysautonomia [2-6,8,9].

Lennon and colleagues discovered a biomarker for NMO; NMO-IgG is an autoantibody initially detected in the serum of 73% of NMO but less than 5% of CMS patients [10], which binds to aquaporin-4 (AQP4) [11], the most abundant water channel in the CNS [12-14]. Differences in serological (NMO-IgG seropositivity rates), clinical, neuroradiological and histological characteristics between NMO and CMS have been described, which support the idea that NMO is distinct from CMS [2-6,8-10,15-24]. Detection of NMO-IgG or AQP4 autoantibodies is clinically useful for early diagnosis of NMO and its related spectrum disorders (NMOSD), including single attack or recurrent LETM without ON, and recurrent ON without ATM; and especially early distinction between NMOSD and CMS [3]. This is critical for proper long-term treatment of patients, as prompt initiation of immunosuppressive medications such as azathioprine with corticosteroid is indicated in NMOSD to prevent relapse whereas immunomodulatory therapies such as beta-interferon and natalizumab may be indicated in CMS [3,7,25-28].

NMO-IgG was first identified by indirect immunofluorescence assay (IIFA) using mouse cerebellum as substrate [10]. Subsequently, other semiquantitative and quantitative techniques have been reported for detection of AQP4 autoantibodies in serum and cerebrospinal fluid (CSF) of NMOSD patients including radioimmunoprecipitation [29], fluorescent-based immunoprecipitation assay (FIPA) [30,31], enzyme-linked immunosorbant assay (ELISA) [32], western blot [33] and cell-based IIFA [30,34]. AQP4 autoantibodies seropositivity rates are reported to be higher by IIFA using transfected mammalian cells which express human AQP4 on their cell membranes (cell-based IIFA) than by IIFA using mammalian CNS tissue (tissue-based IIFA). The optimal assay to detect AQP4 autoantibodies for efficient large-scale clinical service has not been ascertained. In this communication, we compared AQP4 autoantibodies seropositivity rates detected by tissue-based IIFA and cell-based IIFA.

## Patients and Methods

### Patients

Inclusion and exclusion criteria, treatments, follow-up and assessment of patients were as described previously [8] except that in this study, we recruited Hong Kong Chinese patients with CNS IDD of duration 2 years or longer after clinical onset (instead of 4 years of longer as in the previous study) who consented to study. NMO was diagnosed according to revised Wingerchuk criteria [35]. Classical MS was diagnosed according to revised McDonald criteria [36]. A total of 128 patients with CNS IDD consisted of patients with CMS (40), a single attack of acute myelitis (25, two had LETM), NMO (18), relapsing myelitis (15, twelve had LETM), a single attack of ON (14), relapsing ON (9) and a single attack of acute disseminated encephalomyelitis (ADEM) (7). In addition, 35 patients with other neurological diseases consisting of autoimmune myasthenia gravis (18), Guillain-Barre syndrome (5), chronic inflammatory demyelinating polyradiculoneuropathy (5), paraneoplastic neurological disorders (4), Lambert-Eaton myasthenic syndrome (1), polymyositis (1) and systemic lupus erythematosus (1); and ten healthy subjects were studied. Ninety-five of the 128 patients with CNS IDD (30 CMS, 20 single attack of acute myelitis, 10 relapsing myelitis, 10 NMO, 11 single attack of ON, 9 recurrent ON, 5 single attack of ADEM) were studied previously [8]. Treatment of patients were as described previously [8]; in addition, NMOSD patients who did not tolerate azathioprine were treated with mycophenolate mofetil and two NMO patients have been treated with rituximab for fulminant disease (375 mg/m^2 ^weekly for 4 weeks every 6 months) [26,27]. Poor visual outcome was defined as a Snellen visual acuity ≤ 3/60 or a visual field < 20 degrees upon latest assessment by an ophthalmologist. Poor neurological outcome was defined as an EDSS score of ≥ 6.0 (not affected by recent relapse) upon latest assessment by a neurologist.

### Tissue-based indirect immunofluorescence assay for NMO-IgG

Tissued-based IIFA was performed using slides containing monkey cerebellum (The Binding Site, Birmingham, UK) as described previously [8].

### Transfection of human embryonic kidney (HEK) 293 cells with a construct containing the human aquaporin-4 gene

**Figure 1:**
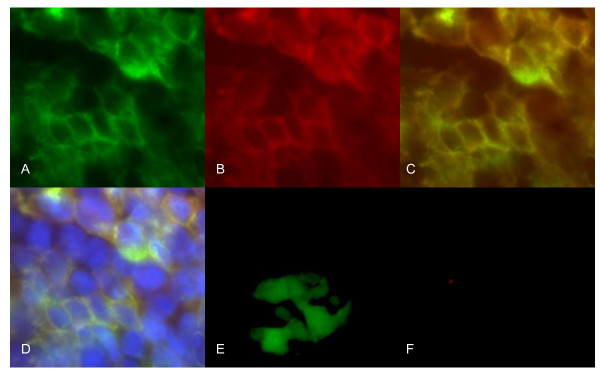
**Indirect immunofluorescence of HEK293 cells transfected for expression of human aquaporin-4 (AQP4)**. A) HEK293 cells were transfected with the human aquaporin-4 (AQP4) gene and show green fluorescence at the cell membrane due to expression of green fluorescent protein (GFP)-AQP4 fusion protein at cell membranes. B) indirect immunoflurescence of these cells, using rabbit anti-human AQP4 antibody (Santa Cruz, CA) as the primary antibody and goat anti-rabbit antibody conjugated with rhobdamine, shows red fluorescence at the cell membrane due to expression of human AQP4 at cell membrane. C) overlap of A and B shows yellow fluorescence due to co-localization of GFP and AQP4 in cell membrane; and D) overlap of image C with staining for cell nuclei by DAPI clearly reveals AQP4 expression at cell membranes. E) HEK293 cells were transfected with empty vector and show green fluorescence over cell cytoplasm due to expression of GFP which, in the absence of AQP4, is not anchored in the cell membrane; and F) indirect immunofluorescence of these cells with rabbit anti-human AQP4 antibody reveals no red fluorescence, consistent with a lack of expression of AQP4.

**Figure 2:**
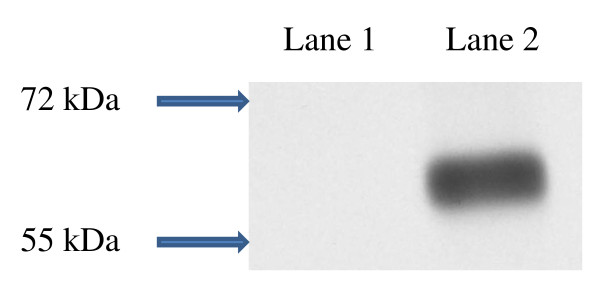
**Western blot analysis of lysate of HEK293 cells, transfected for expression of human aquaporin-4 (AQP4), using rabbit anti-human AQP4 antibody**. Lane 1 contains lysate of HEK293 cells transfected with empty vector and shows no band. Lane 2 contains lysate of HEK293 cells transfected with human AQP4 gene and shows a band of ~64 kDa, consistent with the molecular weight of the green fluorescent protein-AQP4 fusion protein.

We transfected human embryonic kidney (HEK) 293 cells to express a fusion protein composed of full length human AQP4 fused at its N-terminus with green fluorescent protein (GFP) as described by Lennon and colleagues [11]. HEK293 cells express a dystroglycan complex which allows stable insertion of AQP4 into the plasma membrane. Full-length human AQP4 was amplified from an adult human brain cDNA library (BD Biosciences) by PCR using the following primers: forward 5'-GACGGTACCCCATGAGTGACAGACCCAC-3', and reverse 5'-TCCCCCGGGGGATCATACTGAAGACAATA-3' and cloned into a pEGFP-C2 vector (Clontech Laboratories, Inc). HEK293 cells were obtained from ATTC, and cultured in Dulbecco's Modified Eagle Medium (DMEM) in 5% CO_2 _at 37°C. HEK293 cells were transfected with a vector carrying human AQP4 gene or a control vector without human AQP4 by Lipofectamine 2000 (Invitrogen, Carlsbad, CA, USA) according to instructions from the manufacturer. Cells were examined under a microscope for green fluorescence over cell membranes from expression of the GFP-AQP4 fusion protein, and stably transfected cells were selected by G418 (Invitrogen) at 1200 μg/ml. Transfection was confirmed by sequencing, and the presence of GFP-AQP4 fusion protein expression was confirmed by indirect immunofluorescence (Figure 1) and by western blot analysis of cell lysate (Figure 2) using rabbit anti-human AQP4 antibody (Santa Cruz, CA). Control cells were HEK293 cells stably transfected with empty vector without human AQP4 gene. Green fluorescence was observed in the cytoplasm of these cells but not on cell membranes (Figure 1) and western blot analysis revealed no band corresponding to the molecular weight of the GFP-AQP4 fusion protein (Figure 2).

### Cell-based indirect immunofluorescence assay for AQP4 autoantibodies

**Figure 3:**
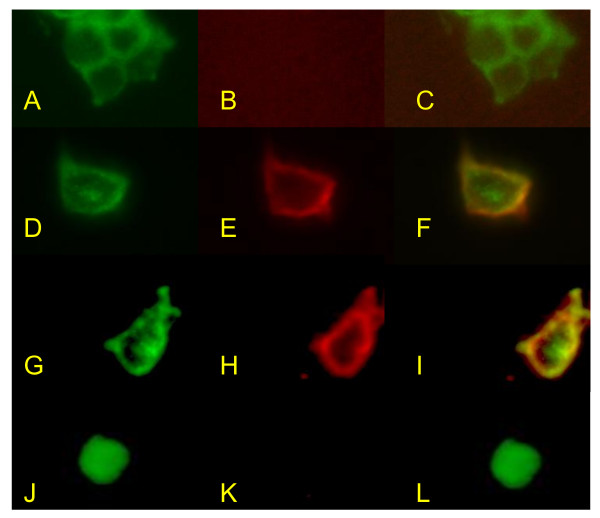
**Cell-based IIFA to detect AQP4 autoantibodies using transfected HEK293 cells that express human AQP4**. A-C, HEK293 cells transfected with human AQP4 gene reveal green fluorescence on cell membranes due to expression of GFP-AQP4 fusion protein as a membrane protein (A). IIFA of serum from a healthy subject (negative control), using secondary goat anti-human IgG conjugated with rhobdamine, shows no red fluorescence (B) signifying seronegativity for AQP4 autoantibodies. With B overlapped with A, no yellow fluorescence is observed (C). D-F, IIFA of transfected cells expressing GFP-AQP4 fusion protein (D) with rabbit anti-human AQP4 antibody as primary antibody (positive control) reveals red fluorescence at the cell membrane (E). When E is overlapped with D, yellow fluorescence is observed (F) signifying colocalization of AQP4 with GFP at cell membranes. G-I, IIFA using transfected HEK293 cells expressing GFP-AQP4 fusion protein (G) of serum from a NMO patient seropositive for NMO-IgG shows red fluorescence at the cell membranes (H). When H is overlapped with G, yellow fluorescence due to colocalization of GFP with AQP4 is observed at cell membranes (I) signifying seropositivity for AQP4 autoantibodies. J-L, IIFA of serum of the same NMO patient in G-I using HEK293 cells transfected with empty vector without human AQP4 gene. The transfected HEK293 cells show green fluorescence in their cytoplasm due to expression of GFP, but in the absence of GFP-AQP4 fusion protein, GFP is not expressed as a membrane protein (J); and IIFA of the patient's serum, seropositive for NMO-IgG, reveals no red fluorescence at cell membranes (K) and no yellow fluorescence when overlapped with J (L). This proves that the human IgG bound to transfected HEK293 cell membrane in H and I represents autoantibodies targeting human AQP4.

Stably transfected HEK293 cells expressing GFP-AQP4 fusion protein or HEK293 cells stably transfected with empty vector were seeded into 8-well chamber slides (Lab Tek, USA), 10,000 cells per well, and incubated in 5% CO_2 _at 37°C for 36-40 hr. Culture medium was removed by gentle suction and the cells were washed one time with phosphate buffered saline (PBS) under gentle shaking for 3 min, and then fixed with 4% paraformaldehyde at room temperature (RT) for 10 min. Cells were washed two times with PBS under gentle shaking for 3 min and then blocked with PBS containing 1% bovine serum albumin (BSA) at RT for 1 hr. Serum of patients and of healthy subjects (negative controls) were diluted at 1:20 with PBS containing 1% BSA, and that of positive controls (rabbit anti-human AQP4 antibody [Santa Cruz, CA]) was diluted at 1:500 with PBS containing 1% BSA. Diluted serum was incubated with rat liver powder at RT for 1 hr to remove non-specific antibodies, then centrifuged at 14,000 rpm for 15 min. 100 μL diluted serum was added to each well and incubated at 4°C overnight. Cells were then washed 3 times with PBS as above, and then incubated with secondary antibody (goat anti-human IgG conjugated with rhobdamine [Invitrogen, Carlsbad, CA, USA], diluted 1:3000 with PBS containing 1% BSA for human serum; goat anti-rabbit IgG conjugated with rhobdamine [Invitrogen], diluted 1:1000 with PBS containing 1% BSA for positive control) for 1 hr at RT in the dark. Cells were then washed 3 times with PBS, mounted with slow-fade gold antifade reagent with DAPI (Invitrogen) and coverslips applied. Slides were examined independently by two investigators (KHC and JSCK) who were blinded to the clinical and laboratory information of studied patients, except for the positive and negative controls, under a fluorescence microscope (Zeiss, Gottingen, Germany) for green and then red fluorescence on cell membranes, and their colocalization on the cell membrane (yellow fluorescence) using Axio Vision software. The presence of green fluorescence on cell membranes in the absence of red fluorescence, or the presence of red fluorescence that did not colocalize with green fluorescence, was scored as negative for AQP4 autoantibodies (-). The presence of red fluorescence on cell membranes that colocalized with green fluorescence was scored as positive for AQP4 autoantibodies and graded on a 3-point scale: weakly positive (+), positive (++) and strongly positive (+++) (Figure 3). Each serum was assayed 3 times and a final score calculated as the median of the 6 scores from the two investigators. Negative serum was restudied at a dilution of 1:5. Negative controls in each experiment included sera from two healthy subjects and positive controls included rabbit anti-human AQP4 antibody (Santa Cruz, CA) as mentioned above and two NMO patients seropositive for NMO-IgG as confirmed by the Clinical Neuroimmunology Laboratory of Mayo Clinic, Rochester, Minnesota. These two NMO-IgG positive sera were positive for AQP4 autoantibodies on this cell-based IIFA (1 positive, 1 strongly positive) but negative on assay using HEK293 cells transfected with empty vector, confirming that they contained autoantibodies targeting human AQP4 (Figure 3).

### Statistical analysis

Seropositivity rates for AQP4 autoantibodies by cell-based IIFA were compared to seropositivity rates for NMO-IgG by tissue-based IIFA for each group of NMOSD patients by the McNemar test to assess statistical significance of differences in sensitivity between the two assays. Clinical, serological and radiological characteristics of NMOSD patients seropositive for AQP4 autoantibodies by cell-based IIFA were compared between those seropositive and seronegative for NMO-IgG by tissue-based IIFA using Chi-square test, student's t-test and Fisher exact test. A p value < 0.05 was considered statistically significant.

## Results

A total of 128 patients with CNS IDD consisted of patients with CMS (40), a single attack of acute myelitis (25, two had LETM), NMO (18), relapsing myelitis (15, twelve had LETM), a single attack of ON (14), relapsing ON (9) and a single attack of acute disseminated encephalomyelitis (ADEM) (7) were studied. In addition, 35 patients with other neurological diseases consisting of autoimmune myasthenia gravis (18), Guillain-Barre syndrome (5), chronic inflammatory demyelinating polyradiculoneuropathy (5), paraneoplastic neurological disorders (4), Lambert-Eaton myasthenic syndrome (1), polymyositis (1) and systemic lupus erythematosus (1); and ten healthy subjects were studied. Table 1 summarizes the demographic and clinical characteristics of the patients with NMO and relapsing myelitis. Table 2 summarizes the seropositivity rates of the various groups for NMO-IgG by tissue-based IIFA and AQP4 autoantibodies by cell-based IIFA. None of the sera negative for AQP4 autoantibodies by cell-based IIFA at 1:20 dilution were positive on repeated assay at 1:5 dilution. Remarkably, 78% of NMO patients were seropositive by cell-based IIFA versus 61% by tissue-based IIFA (p = 0.250), 60% of patients having relapsing myelitis were seropositive by cell-based IIFA versus 40% by tissue-based IIFA (p = 0.250), 75% of patients having relapsing myelitis with LETM were seropositive by cell-based IIFA versus 50% by tissue-based IIFA (p = 0.250). In addition, 33% of patients with relapsing ON were seropositive by cell-based IIFA versus 22% by tissue-based IIFA (p = 1.000), and 14% of patients with a single attack of ON were seropositive by cell-based IIFA versus 7% by tissue-based IIFA (p = 1.000). All patients seropositive for NMO-IgG by tissue-based IIFA were also seropositive for AQP4 autoantibodies by cell-based IIFA. However, the differences in sensitivity between the two assays were not statistically significant for any groups of CNS IDD patients by the McNemar test.

**Table 1:** Clinical and neuroradiological characteristics of patients with neuromyelitis optica and relapsing myelitis.

	NMO (n = 18)	RM (n = 15)
**Female (%)**	15 (83%)	15 (100%)

**Mean onset age in years (range)**	40.1 (18-64)	45.7 (17-70)

**Mean duration of follow-up in years (range)**	6.8 (2-16)	5.1 (1-12)

**Mean number of AM attack (range)**	3.1 (2-9)	2.7 (2-5)

**Patients with LETM (%)**	17 (94%)	12 (80%)

**Mean number of ON attacks (range)**	1.9 (1-6)	not applicable

**Mean relapse rate in first 2 years in number of attack per year (range)**	1.5 (0.5-4.0)	0.8 (0.3-2.0)

**Mean length of MRI T2W hyperintense signal abnormalities in number of vertebral segments (range)**	4.9 (1-16)	4.2 (1-10)

**Patients with MRI brain lesions compatible with inflammatory demyelination (%)**	10 (56%)	6 (40%)

**Patients with CSF OCB (%)**	3 (17%)	3 (20%)

**Patients with other autoimmune disorders or autoantibodies (%)**	9 (50%)	7 (47%)

**Patients with poor visual outcome (%)**	7 (39%)	not applicable

**Mean EDSS score at latest follow-up (range)**	5.9 (2.0-10)	5.4 (1.0-10)

**Patients with poor neurological outcome (%)**	12 (67%)	10 (67%)

**Mortality**	2 (11%)	2 (13%)

Abbreviations: n = number of patients, NMO = neuromyelitis optica, AM = acute myelitis, RM = relapsing myelitis, LETM = longitudinally extensive transverse myelitis, MRI = magnetic resonance imaging, CSF = cerebrospinal fluid, OCB = oligoclonal bands, EDSS = Expanded Disability Status Scale.

**Table 2:** Seropositivity rates for NMO-IgG and AQP4 autoantibodies in different groups of central nervous system idiopathic inflammatory demyelinating disorders, other neurological disorders, and healthy subjects.

Patient/control groups	Number of subjects	Number seropositive for NMO-IgG by tissue-based IIFA (%)	Number seropositive for AQP4 autoantibody by cell-based IIFA (%)	Significance p value
NMO	18	11 (61%)	14 (78%) [3 +++, 3 ++, 8 +]	ns

Relapsing myelitis	15	6 (40%)	9 (60%) [4 ++, 5 +]	ns

Relapsing myelitis with LETM	12	6 (50%)	9 (75%) [4 ++, 5 +]	ns

Single attack of acute myelitis	25	0	1 (4%) [+]	ns

Single attack of LETM	2	0	1 [+]	ns

Relapsing ON	9	2 (22%)	3 (33%) [2 ++, 1 +]	ns

Single attack of ON	14	1 (7%)	2 (14%) [1 ++, 1 +]	ns

Classical MS	40	0	0	ns

ADEM	7	0	0	ns

Healthy subjects	10	0	0	ns

Other neurological disorders	35	0	0	ns

Abbreviations: NMO = neuromyelitis optica, LETM = longitudinally extensive transverse myelitis, ON = optic neuritis, MS = multiple sclerosis, ADEM = acute disseminated encephalomyelitis, IIFA = indirect immunofluorescence, AQP4 = aquaporin-4, +++ strongly positive, ++ positive, + weakly positive, ns = not significant.

Hence, a total of 29 NMOSD patients seropositive for AQP4 autoantibodies were identified by cell-based IIFA, and 20 of these 29 patients (69%) were seropositive for NMO-IgG by tissue-based IIFA. The 9 patients who were seropositive for AQP4 autoantibodies by cell-based IIFA while seronegative for NMO-IgG by tissue-based IIFA included patients with NMO (3), relapsing myelitis with LETM (3), a single attack of LETM (1), relapsing ON (1) and a single attack of ON (1); five of the 9 scored weakly positive (+) and the remaining 4 scored positive (++) on cell-based IIFA. Table 3 summarizes the demographic and clinical characteristics of the 23 NMO and RM patients seropositive for AQP4 autoantibodies by cell-based IIFA and compares those seropositive (n = 17) and seronegative (n = 6) for NMO-IgG by tissue-based IIFA. There was no difference in clinical and neuroradiological characteristics between patients seropositive and seronegative for NMO-IgG by tissue-based IIFA.

**Table 3:** Clinical and neuroradiological characteristics of 23 patients with neuromyelitis optica or relapsing myelitis who are seropositive for AQP4 autoantibodies by cell-based IIFA, divided into those who are seropositive and seronegative for NMO-IgG by tissue-based IIFA.

	NMO IgG +ve (n = 17)	NMO-IgG -ve (n = 6)	Significance p value
**Female (%)**	15 (88%)	6 (100%)	ns

**Mean onset age in years (range)**	42.0 (18-64)	52.8 (17-70)	ns

**Mean duration of follow-up in years (range)**	6.6 (2-15)	3.9 (2-8)	ns

**Patients with LETM (%)**	16 (94%)	6 (100%)	ns

**Mean myelitis attack rate in no. of attacks per year (range)**	0.8 (0.2-2.0)	0.7 (0.3-1.0)	ns

**Mean myelitis attack rate in first 2 years of disease in no. of attacks per year**	1.6 (0.5-3.0)	1.4 (0.5-4.0)	ns

**Mean length of MRI T2W hyperintense signal abnormalities in no. of vertebral segments (range)**	5.3 (2-17)	5.7 (3-10)	ns

**Patients with MRI brain lesions compatible with inflammatory demyelination (%)**	10 (59%)	3 (50%)	ns

**Patients with CSF OCB (%)**	2 (12%)	0	ns

**Patients with other autoimmune disorders or autoantibodies (%)**	5 (29%)	1 (17%)	ns

**Patients with poor visual outcome (%)**	4 (24%)	1 (17%)	ns

**Mean EDSS score at latest follow-up (range)**	5.9 (2.0-10)	6.8 (6.0-8.0)	ns

**Patients with poor neurological outcome (%)**	11 (65%)	6 (100%)	ns

Abbreviations: NMO = neuromyelitis optica, AM = acute myelitis, LETM = longitudinally extensive transverse myelitis, MRI = magnetic resonance imaging, CSF = cerebrospinal fluid, OCB = oligoclonal bands, EDSS = Expanded Disability Status Scale, ns = not significant.

## Discussion

Our results are consistent with other reports confirming that both NMO-IgG detected by tissue-based IIFA and AQP4 autoantibodies detected by cell-based IIFA are specific for NMOSD, and that their detection facilitates early distinction of NMOSD from CMS [5,8,10,15]. The original IIFA using mouse cerebellum described by Lennon and colleagues is 73% sensitive in detection of NMO-IgG and 91% specific for NMO, and is 46% sensitive for high-risk syndrome (one or more attacks of LETM without cerebral lesions that satisfy criteria for MS, or recurrent ON) [10]. The reported sensitivity rates of tissue-based IIFA to detect autoantibodies binding to mouse, rat or monkey astrocytic AQP4 range from 19% to 73% [5,8,10,15-21].

Transfected HEK 293 cells express human AQP4 homotetramers closely packed on cell membranes [37], and allow detection of AQP4 autoantibodies binding to extracellular epitopes of human AQP4. Our results show that cell-based IIFA detects AQP4 autoantibodies in 78% of NMO patients and 75% of patients having relapsing myelitis with LETM, while tissue-based IIFA detect NMO-IgG in 61% of NMO patients and 50% of patients having relapsing myelitis with LETM. In addition, cell-based IIFA detects AQP4 autoantibodies in 33% of relapsing ON patients while tissue-based IIFA detects NMO-IgG in 22% of this group. These results suggest that cell-based IIFA is more sensitive than tissue-based IIFA in detection of IgG AQP4 autoantibodies in NMOSD. Takahashi and colleagues reported that 91% of NMO and 85% of high-risk syndrome (recurrent LETM) patients are seropositive for AQP4 autoantibodies detected by a similar cell-based IIFA, with a specificity of 100% [34]. In addition, among their 21 NMO patients seropositive for AQP4 autoantibodies by cell-based IIFA, patients seropositive for NMO-IgG by tissue-based IIFA (15 patients) had a higher frequency of longitudinally extensive spinal cord lesions on MRI than patients seronegative for NMO-IgG by tissue-based IIFA (6 patients), 100% versus 50% (p = 0.015) [34]; this was not observed in our patients. Waters and colleagues studied AQP4 autoantibodies in NMO and LETM patients by tissue-based IIFA, FIPA and a similar cell-based IIFA; and reported that 14 of 24 NMO patients (58%) were seropositive by tissue-based IIFA, 19 of 25 NMO patients (76%) seropositive by FIPA and 20 of the 25 NMO patients (80%) seropositive by cell-based IIFA. In addition, 5 of 10 LETM patients (50%) were seropositive by tissue-based IIFA and 6 of 11 LETM patients (55%) seropositive by both FIPA and cell-based IIFA [30]. Our results are consistent with those of Waters and colleagues.

McKeon and colleagues compared sensitivities and specificities of tissue-based IIFA and FIPA and reported that the sensitivity rates for NMO are 58% by tissue-based IIFA, 33% by FIPA and 63% by combining the two assays, while the sensitivity rates for relapsing LETM are 29% by tissue-based IIFA, 6% by FIPA and 29% by combined assays; and specificity rates for NMO and relapsing LETM are 99.6% by tissue-based IIFA, 99.3% by FIPA and 99.2% by combined assays [31]. In addition, among 331 patients seropositive for AQP4 autoantibodies by FIPA, 76 (23%) were seronegative by tissue-based IIFA. In this large-scale clinical practice-based study from the Mayo Clinic, the investigators concluded that AQP4 autoantibodies detected by tissue-based IIFA or FIPA are highly specific for NMOSD, and that FIPA is significantly less sensitive than tissue-based IIFA, although combined testing improved sensitivity by 5% [31].

In conclusion, cell-based IIFA is slightly more sensitive than tissue-based IIFA in detection of AQP4 autoantibodies. As the cell-based IIFA requires technical expertise in the observation of GFP and AQP4 co-localization, tissue-based IIFA and FIPA may be more useful for clinical service, as it allows rapid large-scale screening for AQP4 autoantibodies, and cases that are serum negative by tissue-based IIFA and FIPA can be further studied for AQP4 autoantibodies by cell-based IIFA if clinical suspicion for NMOSD is high.

## Competing interests

The authors declare that they have no competing interests.

## Authors' contributions

KHC designed the study, recruited patients, performed some of the experiments, interpreted the immunofluorescence results, and drafted the manuscript. JSCK transfected the HEK293 cells with human AQP4 gene and is one of the investigators who interpreted the immunofluorescence results. PWLH was responsible for design of the primers and western blot analysis. JWMH performed PCR and supervised transfection experiments. ACYC was responsible for synthesis of rat liver powder for tissue-based immunofluorescence assay. DBR facilitated tissue-based immunofluorescence assay and supervised western blot analysis. All the authors have read and approved the final version of the manuscript.
